# Dietary behaviour of man-eating lions as revealed by dental microwear textures

**DOI:** 10.1038/s41598-017-00948-5

**Published:** 2017-04-19

**Authors:** Larisa R. G. DeSantis, Bruce D. Patterson

**Affiliations:** 1 Department of Earth and Environmental Sciences, Vanderbilt University, Nashville, TN 37235-1805 USA; 2 Integrative Research Center, Field Museum of Natural History, Chicago, IL 60605-2496 USA

## Abstract

Lions (*Panthera leo*) feed on diverse prey species, a range that is broadened by their cooperative hunting. Although humans are not typical prey, habitual man-eating by lions is well documented. Fathoming the motivations of the Tsavo and Mfuwe man-eaters (killed in 1898 in Kenya and 1991 in Zambia, respectively) may be elusive, but we can clarify aspects of their behaviour using dental microwear texture analysis. Specifically, we analysed the surface textures of lion teeth to assess whether these notorious man-eating lions scavenged carcasses during their depredations. Compared to wild-caught lions elsewhere in Africa and other large feliforms, including cheetahs and hyenas, dental microwear textures of the man-eaters do not suggest extreme durophagy (e.g. bone processing) shortly before death. Dental injuries to two of the three man-eaters examined may have induced shifts in feeding onto softer foods. Further, prompt carcass reclamation by humans likely limited the man-eaters’ access to bones. Man-eating was likely a viable alternative to hunting and/or scavenging ungulates due to dental disease and/or limited prey availability.

## Introduction

Lions (*Panthera leo*) once inhabited much of Africa, southeastern Europe, and southwestern Asia^1^. Currently, lions (*Panthera leo*) occupy savannas and deserts in sub-Saharan Africa (excluding rainforests and the Sahara), with an isolated population located in the Gir Forest of India. They are highly social, and males and females each live in persistent bonded groups^2^. Lion behaviours, diets, and social groupings all vary enormously in response to spatial or temporal shifts in prey availability and habitat structure^3^. Smaller groups and females prey mainly on zebra and wildebeest whereas larger groups and males feed differentially on buffalo^4, 5^. Lions are known to consume a diverse suite of prey with preferences for gemsbok, buffalo, wildebeest, giraffe, zebra, Thomson’s gazelle, warthog, kongoni, and topi^4, 6^. Further, habitat and droughts can affect lion preferences and prey vulnerability to predation, with lions increasing the proportion of elephant calves consumed during droughts^7^. Their current range collapse, to 20% of historic values, is driven not by limited adaptability but rather by habitat loss and fragmentation, prey depletion, and direct persecution^8^.

Man-eating, or consumption of humans as women and children are often victims, has occasionally been a dietary strategy of lions and other pantherines^9, 10^. Two notorious lions (popularized in the 1996 film *The Ghost and the Darkness*) terrorized people near Tsavo by repeatedly killing and consuming railway workers in 1898, and one from Mfuwe, Zambia consumed six people as recently as 1991^11^. Colonel J. H. Patterson, who eventually killed the Tsavo man-eaters in December 1898, estimated that they had killed and eaten 135 people^12^. However, stable isotope analysis of their hair and bone collagen suggests that they had consumed ~35 people, representing roughly 30% of the first man-eater’s diet (FMNH 23970) and ~13% of the second man-eater’s diet (FMNH 23969)^13^. The reasons for the lions’ differential reliance on humans, and for man-eating by lions in general, remain unclear. Many hypotheses have been proposed regarding the motivations of the man-eating lions, including an extended drought, a 1898 rinderpest outbreak that ravaged prey populations, various cultural causes, and/or dental disease^11, 14, 15^.

**Figure 1:**
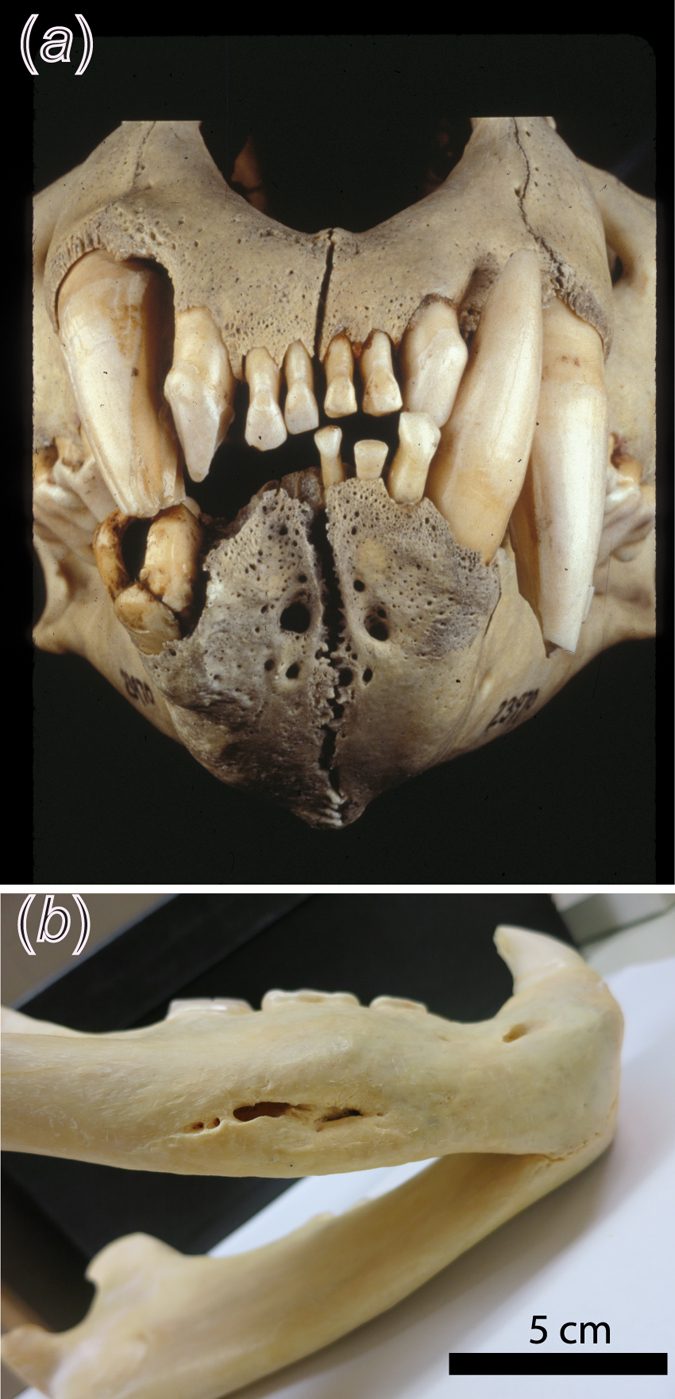
Images of injuries to Tsavo’s 1st man-eater (**a**), FMNH 23970 and the Mfuwe man-eater (**b**), FMNH 163109. Image (**a**), Field Museum of Natural History image Z-94320_11c by John Weinstein documents a broken lower right canine (which had a periapical abscess) and loss of the lower three right incisors - presumably from the kick of a struggling prey - and subsequent over-eruption of the upper right incisors and rotation of the upper right canine both labially and mesially in the absence of the interlocking lower canine. In (**b**), multiple oval-shaped intraosseous lesions are visible on the right mandible, superficial to an occluded mandibular canal and associated with a chronically draining fistula^15^. Again, these injuries are consistent with blunt trauma from a powerful ungulate kick.

Evidence of dental disease is quite clear in two of the three man-eating lions. One lion (the first Tsavo man-eater), with a broken canine, developed a periapical abscess and lost three lower right incisors^15^ (see Fig. 1). The pronounced toothwear and extensive cranial remodeling suggests that the lion had broken his canine several years earlier^15^. The second Tsavo man-eater had minor injuries including a fractured upper left carnassial and subsequent pulp exposure, although these types of injuries are fairly common and were unaccompanied by disease^16^. Similarly, the man-eater from Mfuwe had fractured its right mandibular ramus. These injuries may have been decisive factors influencing their consumption of humans. While it is difficult to assess the motivations of the man-eating lions, we can clarify aspects of their behaviour prior to their death. Most notably, we can assess if their circumstances caused them to rely more heavily on scavenging carcasses shortly before they were killed, as suggested by contemporary reports of the sounds of bone-crunching on the edge of camp^12^. Dental microwear texture analysis (DMTA) can clarify the textural properties of consumed food, including durophagy in carnivorans, and clearly distinguishes feliforms that eat primarily flesh (the cheetah, *Acinonyx jubatus*), from generalists (*P. leo*), and various hyenas which are known to fully consume carcasses, including bone^17, 18^.

In contrast to two-dimensional dental microwear, which relies on human identification and counting of microscopic wear features such as pits and scratches, 3D DMTA quantifies surfaces using scale-sensitive fractal analysis^19–22^. Complexity (*Asfc*) distinguishes taxa that consume brittle foods from taxa that consume softer ones^19–23^. Anisotropy (*epLsar*), the degree to which features share similar orientations, instead indicates tough food consumption when values are high^19–23^. Textural fill volume (*Tfv*) is a measure of the difference in volume filled by large (10 µm) and small (2 µm) diameter square cuboids; high values indicate many deep features between these sizes^21, 23^. For extant carnivorous taxa, increased complexity and increased textural fill volume are associated with increased durophagy^17, 18, 23–26^.

Here, we compare dental microwear attributes of man-eating lions from Tsavo (from 1898) and from Mfuwe (from 1991) to wild-caught lions from throughout their range to assess if the dental microwear textures of man-eaters suggest extreme durophagy shortly before death. A secondary aim is to improve our understanding of how age, sex, and body size may influence access to carcasses in wild-caught lions. We test the hypothesis that the diets of man-eating lions consisted primarily of hard objects (e.g. bone), and that these lions mainly engaged in scavenging (perhaps out of desperation) prior to their death.

## Results

**Figure 2:**
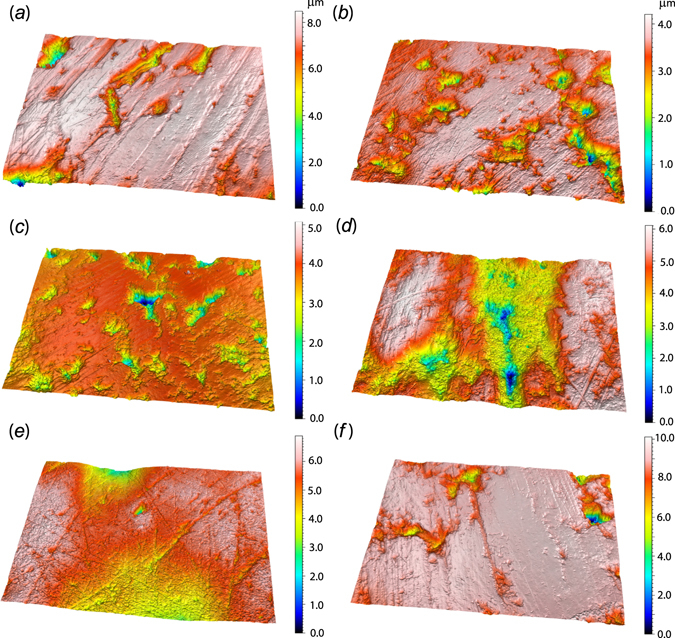
Digital elevation models of microwear surfaces of (**a**,**b**) wild-caught lions (FMNH 20762; FMNH 33479), (**c**) a captive lion (FMNH 54639), and (**d**–**f**) man-eating lions (Tsavo 1^st^ man-eater, FMNH 23970; Tsavo 2^nd^ man-eater, FMNH 23969; and Mfuwe man-eater, FMNH 163109). All models noted here represent 204 × 274 μm in area with relevant z-scale bars noted for each image (μm).

**Figure 3:**
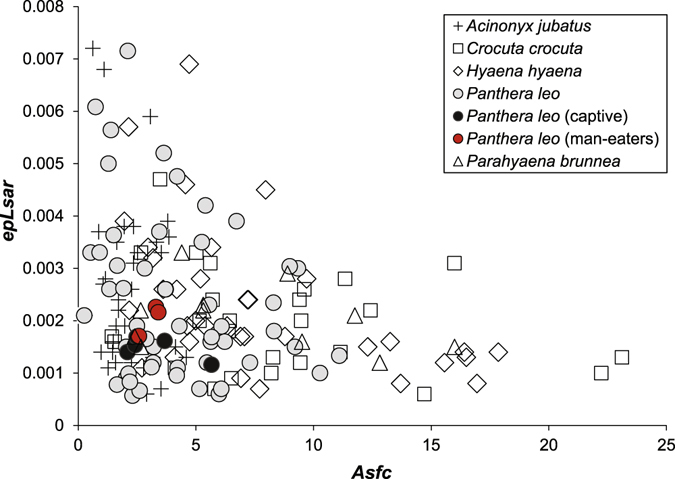
Bivariate plot of anisotropy (*epLsar*) and complexity (*Asfc*) of cheetahs, hyenas (multiple species), captive lions, man-eating lions, and wild-caught lions.

Results are illustrated in Figs 2 and 3 and summarized in Table 1 (all primary data are included in electronic supplementary materials, Supplemental Tables 1 and 2). As previously documented, complexity values of *A. jubatus* (median = 2.180) are significantly lower than for *P. leo* (p < 0.001) and all hyenas (p-values are all < 0.0001)^18^. *P. leo* (excluding the man-eaters) have complexity values that range from 0.258 to 11.096 (median = 3.669; Table 1) and are significantly lower than all hyenas (median values range from 5.316 in *Parahyaena brunnea*, 6.328 in *Hyaena hyaena*, to 7.354 in *Crocuta crocuta*; p-values are all < 0.05). Anisotropy values are indistinguishable between all feliform species here examined (as noted in prior work^18^). Textural fill volume of wild-caught feliforms is lowest in *A. jubatus*, followed by *P. leo*, with *A. jubatus* and *P. leo* having significantly lower *Tfv* values than all hyenas (p < 0.05). The captive lions have the lowest *Tfv* values (median = 3.486).

**Table 1:** Descriptive statistics for each DMTA variable of *Panthera leo* by category (captive, man-eater, wild-caught).

Taxon	Statistic	*n*	*Asfc*	*epLsar*	*Tfv*
*Panthera leo*	Median	5	2.481	0.0015	3486
(captive)	Mean		3.266	0.0015	5165
	Standard Deviation		1.469	0.0002	4187
	Minimum		2.092	0.0012	1930
	Maximum		5.667	0.0017	11952
	Total Range		3.574	0.0005	10022
*Panthera leo*	Median	3	3.307	0.0022	9360
(man-eating)	Mean		3.097	0.0020	9280
	Standard Deviation		0.450	0.0003	3185
	Minimum		2.581	0.0017	6055
	Maximum		3.403	0.0023	12424
	Total Range		0.823	0.0006	6369
Panthera leo*	Median	52	3.669	0.0019	6812
(wild-caught)	Mean		4.229	0.0024	7802
	Standard Deviation		2.712	0.0016	5544
	Minimum		0.258	0.0006	151
	Maximum		11.096	0.0072	20445
	Total Range		10.838	0.0066	20294

Comparative data of other extant feliforms were published previously^18^. *n*, number of individuals sampled; *Asfc*, area-scale fractal complexity; *epLsar*, anisotropy; *Tfv*, textural fill volume. All teeth analyzed are lower first molars, per Materials and Methods. *Denotes the inclusion of data (n = 29) from a prior study^18^.

The man-eating lions have DMTA values (*Asfc*, *epLsar*, and *Tfv*) indistinguishable from other wild-caught *P. leo* (all p-values > 0.69; Tables 1 and S1; Fig. 2), and captive and man-eating lions have nearly identical mean *Asfc* values (3.266 and 3.097, respectively). All man-eater *Asfc* values (ranging from 2.581 to 3.403) fall below the mean and median values for all hyenas^18^. Man-eaters are indistinguishable from all extant taxa in all DMTA attributes (likely due to their limited sample size, n = 3); however, statistical comparisons of *Asf*c values of man-eating lions to *C. crocuta* and *H. hyaena* (p = 0.062, p = 0.102, respectively) identify marginally significant differences in dietary behavior (all p-values ≤ 0.10). Further, man-eating lion *Asfc* values range from 2.581 to 3.403 while 83% of all hyena *Asfc* values (all three species combined) and 88% of *C. crocuta Asfc* values exceed the highest man-eating lion *Asfc* value of 3.403. In contrast, 83% of *A. jubatus* specimens have *Asfc* values less than 3.403 (and all are < 4.6).

Wild-caught male and female lions are indistinguishable in all DMTA attributes (including and excluding the male man-eaters), although females have significantly greater variance of *epLsar* (Levene’s median test, p = 0.016, p = 0.042, respectively; Supplemental Table 1). Correlations between all DMTA attributes and skull measurements (indicative of body size) in a subset of lions were not significant (Supplemental Table 2). However, age was negatively correlated with *Tfv* in wild-caught lions (only when excluding the man-eaters from analyses; Pearson’s correlation co-efficient = −0.512; p = 0.036).

## Discussion

Wild-caught lions not known to hunt humans have highly variable complexity values (total range of 10.838; Table 1). Females actively hunt and take down prey while coalition males often gain preferential access to fresh kills^2^. While males and females don’t differ in mean values for any DMTA attributes here examined, females do have significantly greater variance of anisotropy (with values ranging from 0.0006 to 0.0072 in females as compared to 0.0006 to 0.0048 in males). This suggest that some females eat a mixture of flesh and bones while others have access to fresh kills (eating a greater proportion of tougher flesh) consistent with previous work documenting highly variable feeding behavior in lions^3^. Interestingly, body size and age does not appear to dictate durophagous behaviour as inferred from dental microwear textures.

In contrast, DMTA data (most notably low *Asfc* values) suggest that the man-eating lions examined were not fully consuming carcasses prior to their death. Despite Patterson’s colorful accounts of bone-crunching outside the camp at Tsavo^12^, such behaviour of the Tsavo man-eaters is not supported by DMTA.

DMTA attribute values of man-eating lions appear not only typical but overlap in ‘*Asfc*-*eplsar*’ space with those of captive lions, which are typically fed softer foods (e.g. horsemeat, beef^27^). These similarities, in addition to the absence of *Asfc* values close to or exceeding mean and median hyena values (7.946 and 6.474, respectively, when combining all hyena species included in previous work^18^), suggest that, in the final weeks or months of their lives, the man-eating lions consumed softer parts of humans and other prey and did not fully consume carcasses. The absence of bone consumption/processing, as inferred from low man-eating lion *Asfc* values, may have been due to either their own preferences/limitations (potentially due to injury). Further, the reclamation of human carcasses at daybreak before lions could completely consume them, may have played a role in limiting durophagy. However, isotopic studies of the Tsavo man-eaters document that humans comprised a minor component of their prey consumption so that human carcass recovery could only play an ancillary role^13^.

Two of the three man-eaters (FMNH 23970, FMNH 163109; see Fig. 1) had serious infirmities to their jaws and/or canines, potentially hindering consumption of hard food items and/or reduced prey handling ability (prey are seized and held with teeth and jaws). Tooth breakage per se does not produce dietary shifts as most older lions display some sort of wear or breakage to their dentition^28^. However, dental disease is another matter, and incapacitation via an abscessed or a fractured mandible may have prompted the Tsavo and Mfuwe lions to seek more easily subdued prey. Infirmities such as these were frequently associated with man-eating incidents by tigers and leopards in colonial India^9, 29, 30^.

The second Tsavo lion had less pronounced injuries, consistent with mandibular damage sustained during normal feeding behavior (i.e. a fractured upper carnassial tooth with pulp exposure)^15^. The second man-eating Tsavo lion also consumed a smaller percentage of humans (~13%) than the first man-eating Tsavo lion (~30%) during the last few months of its life (as inferred from stable isotopes in hair)^13^. However, dental injury is fairly common in lions^16, 28^ with 40% of lions from one study^28^ having damaged dentitions. Contrary to expectations, only 23% of “problem lions” in Tsavo East National Park (lions killed by park rangers attacking people or livestock) had dental damage^28^, suggesting that minor to moderate levels of damage unaccompanied by disease does not trigger man-eating or marauding. The second Tsavo lion may have simply shared meals through his social bonds with the first man-eater. Further, it should be noted that bone collagen values (which reflect diet over multiple years) and hair tufts (which reflect consumption during the final months of life) suggest that man-eating behavior varied over the life of the individual lions and both lions may have consumed similar amounts of human prey earlier in their life^13^.

DMTA data here suggests that man-eating lions didn’t completely consume carcasses of humans or ungulates. Instead, humans likely supplemented an already diverse diet^31^. Anthropological evidence suggests that humans were a frequent prey item of leopards and other large felids, which dragged their victims up into trees or down into caves for latter consumption^32–35^. Further, evidence of man-eating by pantherines continues, with more than 563 humans killed between January 1990 and September 2004 by lions in Tanzania^10^. Although lions today seldom hunt humans as compared to other prey species^6^, increasing human populations and declining prey numbers may cause man-eating to become a viable option for lions.

## Materials and Methods

The man-eating lions from Tsavo, Kenya (FMNH 23969, FMNH 23970) and Mfuwe, Zambia (FMNH 163109) were here analysed and compared to extant wild-caught *P. leo* specimens from throughout Africa, with two individuals (AMNH 54995, AMNH 54996) from Gir Forest, India (n = 55; 26 here examined and 29 from previously published work^18^). We also included five captive zoo lions in the analysis, as a separate group, and compared lion groups (captive, man-eaters, and wild-caught) with the following extant feliforms: *Acinonyx jubatus* (cheetah, *n* = 36), *Crocuta crocuta* (spotted hyaena, *n* = 26), *Hyaena hyaena* (striped hyena, n = 35), and *Parahyaena brunnea* (brown hyena, n = 11) from previously published work^18^.

The enamel region of the lower carnassial shearing facet of the m1 trigonid was examined on all specimens as described in prior work^17, 18, 24, 25^. This tooth is used by carnivores both to slice meat and to crush bone. All specimens were scanned on a Sensofar *PLu neox* optical profiler (at Vanderbilt University) in three dimensions in four adjacent fields of view, for a total sampled area of 204 × 276 µm^2^ and subsequently analysed using SSFA software (ToothFrax and SFrax, Surfract Corp., www.surfrait.com) to characterise tooth surfaces according to the following variables: (i) complexity (*Asfc*); (ii) anisotropy (*epLsar*); and, (iii) textural fill volume (*Tfv*)^19–23^.

As most DMTA variables are non-normally distributed, we used non-parametric statistical tests (Kruskal–Wallis and Dunn’s procedure) to compare differences between groups. DMTA attribute values were compared between male and female *P. leo* specimens (Supplemental Table 1) using Mann-Whitney tests. Correlations were also assessed between DMTA values and body size proxies (greatest length of skull, zygomatic width) and age (based on toothwear and suture closure criteria from prior work^36^) in a subset of African lions from which these data were available (Supplemental Table 2).

## Electronic supplementary material

Supplemental Tables 1 and 2
