# Supplementary material for: Dietary behaviour of man-eating lions as revealed by dental microwear textures
Source: Sci Rep. 2017 Apr 19;7:904. doi: 10.1038/s41598-017-00948-5 (PMC5430416; doi:10.1038/s41598-017-00948-5)
Supplement: Supplementary file 1 — Supplemental Tables 1 and 2 [file 41598_2017_948_MOESM1_ESM.pdf]

Dietary behaviour of man-eating lions as revealed by dental microwear textures

Larisa R. G. DeSantis[1] and Bruce D. Patterson[2]

1. Department of Earth and Environmental Sciences, Vanderbilt University, Nashville, TN 37235-1805, USA.

2. Integrative Research Center, Field Museum of Natural History, Chicago, IL 60605-2496, USA

Author for correspondence: Larisa DeSantis, e-mail: larisa.desantis@vanderbilt.edu

Keywords: diet, dental microwear, man-eating, *Panthera leo*, Africa

Supplemental Tables 1-2

Supplemental Table 1. All *Panthera leo* specimens examined, meta-data, and dental microwear textural data.

Status	Museum	ID	tooth	*Asfc*	*epLsar*	*Tfv*	Sex	Locality	Pub.
Captive/Zoo Specimen	FMNH	49340	rm1	2.092	0.0014	2104	F	Zoo	N
Captive/Zoo Specimen	FMNH	54639	lm1	3.671	0.0016	1930	F	Zoo	N
Captive/Zoo Specimen	FMNH	121667	lm1	2.419	0.0015	6355	F	Zoo	N
Captive/Zoo Specimen	FMNH	135278	rm1	5.667	0.0012	11952	F	Zoo	N
Captive/Zoo Specimen	FMNH	173259	rm1	2.481	0.0017	3486	M	Zoo	N
man-eater (Mfuwe)	FMNH	163109	lm1	2.581	0.0017	12424	M	Africa: Zambia	N
man-eater (Tsavo, first)	FMNH	23970	lm1	3.307	0.0023	9360	M	Africa: Kenya	N
man-eater (Tsavo, second)	FMNH	23969	rm1	3.403	0.0022	6055	M	Africa: Kenya	N
wild-caught	AMNH	17274	rm1	6.729	0.0039	11298	U	Africa	P
wild-caught	AMNH	36420	lm1	5.634	0.0016	18224	U	Africa: Kenya	P
wild-caught	AMNH	52072	rm1	3.719	0.0026	20445	M	Africa: Zaire	P
wild-caught	AMNH	52073	rm1	3.635	0.0052	6838	F	Africa: Zaire	P
wild-caught	AMNH	54995	lm1	7.3	0.0012	2342	M	Asia: India (Gir Forest)	P
wild-caught	AMNH	54996	rm1	5.981	0.0006	16602	F	Asia: India (Gir Forest)	P
wild-caught	AMNH	81836	lm1	9.211	0.0015	15766	U	Africa: South Africa	P
wild-caught	AMNH	83410	rm1	10.275	0.001	6761	U	Africa: Central African Republic	P
wild-caught	AMNH	88635	lm1	6.084	0.0007	5452	U	Africa: Kenya	P
wild-caught	AMNH	161732	rm1	5.469	0.0012	11144	F	Africa: Malawi	P
wild-caught	FMNH	1443	lm1	6.091	0.0019	9584	M	Africa: Somalia	N
wild-caught	FMNH	20757	lm1	1.650	0.0008	2460	M	Africa: Kenya	N
wild-caught	FMNH	20758	lm1	3.119	0.0011	1302	F	Africa: Kenya	N
wild-caught	FMNH	20762	lm1	4.204	0.0048	4171	M	Africa: Kenya	N
wild-caught	FMNH	30778	lm1	2.311	0.0006	1099	M	Africa: Sudan	N
wild-caught	FMNH	33479	lm1	2.137	0.0010	927	M	Africa: Tanzania	N
wild-caught	FMNH	33480	rm1	0.738	0.0061	1145	F	Africa: Tanzania	N
wild-caught	FMNH	35131	rm1	2.614	0.0007	2760	M	Africa: Tanzania	N

wild-caught	FMNH	35132	rm1	2.895	0.0017	1935	F	Africa: Tanzania	N
wild-caught	FMNH	35739	rm1	5.669	0.0017	11322	M	Africa: Botswana	N
wild-caught	FMNH	35741	lm1	0.914	0.0033	3333	M	Africa: Botswana	N
wild-caught	FMNH	35742	rm1	1.392	0.0056	8523	F	Africa: Botswana	N
wild-caught	FMNH	35743	lm1	4.188	0.0010	2185	F	Africa: Botswana	N
wild-caught	FMNH	42129	lm1	1.328	0.0026	393	U	Africa: Malawi	N
wild-caught	FMNH	75608	rm1	1.510	0.0036	3659	M	Africa: Kenya	N
wild-caught	FMNH	75609	rm1	2.196	0.0008	886	M	Africa: Kenya	N
wild-caught	FMNH	89926	lm1	8.990	0.0030	3645	M	Africa: Botswana	N
wild-caught	FMNH	127836	rm1	11.096	0.0013	5975	F	Africa: Tanzania	N
wild-caught	FMNH	127838	lm1	2.107	0.0072	11426	F	Africa: Tanzania	N
wild-caught	FMNH	127839	rm1	4.299	0.0019	13533	F	Africa: Tanzania	N
wild-caught	FMNH	127840	lm1	1.660	0.0031	432	U	Africa: Tanzania	N
wild-caught	FMNH	206425	rm1	1.923	0.0026	11551	M	Africa: Zambia	N
wild-caught	FMNH	213656	rm1	8.301	0.0023	14199	F	Africa: Kenya	N
wild-caught	SAM	3983	rm1	3.454	0.0037	12248	M	Africa: Botswana	P
wild-caught	SAM	14893	rm1	5.407	0.0042	12296	U	Africa: South Africa	P
wild-caught	SAM	36873	rm1	1.287	0.005	8322	F	Africa: South Africa	P
wild-caught	SAM	36874	lm1	8.323	0.0018	14470	U	Africa: South Africa	P
wild-caught	SAM	36875	rm1	3.703	0.0026	6180	U	Africa: South Africa	P
wild-caught	SAM	38222	rm1	9.336	0.003	12675	U	Africa: Namibia	P
wild-caught	SAM	39302	rm1	0.525	0.0033	151	F	Africa: Namibia	P
wild-caught	SAM	39870	rm1	2.826	0.003	12955	U	Africa: Namibia	P
wild-caught	USNM	172677	rm1	2.041	0.0015	2362	M	Africa	P
wild-caught	USNM	182297	rm1	4.225	0.0011	6399	M	Africa: Kenya	P
wild-caught	USNM	216601	rm1	0.258	0.0021	1804	U	Africa: Tanzania	P
wild-caught	USNM	216602	rm1	3.203	0.0015	14247	U	Africa: Tanzania	P
wild-caught	USNM	216603	rm1	2.497	0.0019	17066	U	Africa: Tanzania	P
wild-caught	USNM	230920	rm1	3.195	0.0012	10381	U	Africa	P
wild-caught	USNM	236919	rm1	5.252	0.0035	12992	F	Africa: Zambia	P

wild-caught	USNM	236920	rm1	2.04	0.0009	7965	U	Africa: Tanzania	P
wild-caught	USNM	537000	rm1	5.156	0.0007	6785	U	Africa: Kenya	P
wild-caught	USNM	540000	rm1	6.228	0.0016	3815	M	Africa: Kenya	P
wild-caught	USNM	565762	rm1	5.563	0.0023	11271	U	Africa	P

Status, captive, man-eaters, or wild-caught; Museum, AMNH - American Museum of Natural History, FMNH- Field Museum of Natural History, Chicago, IL, SAM-South African Museum in Cape Town, South Africa, USNM - National Museum of Natural History, Smithsonian Institution in Washington D.C., USA; ID, notes museum catalogue number; Tooth, all teeth are lower m1 carnassials with right (r) or left (l) noted; *Asfc*, area-scale fractal complexity; *epLsar*, anisotropy; *Tfv*, textural fill volume; Sex, male (M), female (F), or unknown (U); Locality information; and Pub.-Publication data noting if published previously in Ref. [18] (P) or if new to this paper (N).

Supplemental Table 2. Age (in years) and cranial measurement data (GLS, greatest length of skull; ZW, zygomatic width) for *Panthera leo* specimens from the Field Museum of Natural History (FMNH).

Status	Museum	ID	Age	GLS	ZW
man-eater (Mufuwe)	FMNH	163109	5.0	363.0	223.0
man-eater (Tsavo, first)	FMNH	23970	7.0	353.0	232.0
man-eater (Tsavo, second)	FMNH	23969	7.0	357.0	224.0
wild-caught	FMNH	20757	3.5	346.0	217.5
wild-caught	FMNH	20758	6.5	290.0	186.0
wild-caught	FMNH	30778	5.5	365.0	241.5
wild-caught	FMNH	33479	5.0	363.0	234.5
wild-caught	FMNH	33480	6.0	289.0	198.0
wild-caught	FMNH	35131	7.5	384.0	256.0
wild-caught	FMNH	35132	6.0	304.0	191.0
wild-caught	FMNH	35739	3.5	371.0	231.5
wild-caught	FMNH	35741	10.5	395.0	245.0
wild-caught	FMNH	35742	4.5	296.5	191.5
wild-caught	FMNH	35743	4.5	313.0	199.0
wild-caught	FMNH	75608	5.5	360.0	241.0
wild-caught	FMNH	75609	5.5	375.0	239.0
wild-caught	FMNH	89926	5.5	391.0	236.0
wild-caught	FMNH	127836	6.0	310.0	193.5
wild-caught	FMNH	127838	3.5	287.0	186.0
wild-caught	FMNH	127839	3.0	289.0	181.0
